# Sedentary subjects have higher PAI-1 and lipoproteins levels than highly trained athletes

**DOI:** 10.1186/1758-5996-2-7

**Published:** 2010-01-22

**Authors:** Fabio S Lira, Jose C Rosa, Adriano E Lima-Silva, Hélio A Souza, Erico C Caperuto, Marília C Seelaender, Ana R Damaso, Lila M Oyama, Ronaldo VT Santos

**Affiliations:** 1 Department of Physiology, Division of Nutrition Physiology, Federal University of Sao Paulo, Brazil; 2 Sports Science Research Group, Federal University of Alagoas, Brazil; 3 Molecular Biology of the Cell Group, Institute of Biomedical Sciences, Department of Cell and Developmental Biology, University of São Paulo, Brazil; 4 Department of Bioscience, Baixada Santista Campus, Federal University of São Paulo, Brazil

## Abstract

Physical exercise protects against the development of cardiovascular disease, partly by lowering plasmatic total cholesterol, LDL-cholesterol and increased HDL-cholesterol levels. In addition, it is now established that reduction plasmatic adiponectin and increased C-reactive protein (CRP) and plasminogen activator inhibitor-1 (PAI-1) levels play a role in the maintenance of an inflammatory state and in the development of cardiovascular disease. This study aimed to examine plasma lipid profile and inflammatory markers levels in individual with sedentary lifestyle and/or highly trained athletes at rest. Methods: Fourteen male subjects (sedentary lifestyle n = 7 and highly trained athletes n = 7) were recruited. Blood samples were collected after an overnight fast (~12 h). The plasmatic lipid profile (Triglycerides, HDL-cholesterol, LDL-cholesterol, total cholesterol, LDL-oxidized and total cholesterol/HDL-c ratio), glucose, adiponectin, C - reactive protein and PAI-1 levels were determined. Results: Total cholesterol, LDL-cholesterol, TG and PAI-1 levels were lower in highly trained athletes group in relation to sedentary subjects (p < 0.01). In addition, we observed a positive correlation between PAI-1 and total cholesterol (r = 0.78; p < 0.0009), PAI-1 and LDL-c (r = 0.69; p < 0.006) and PAI-1 and TG levels (r = 0.56; p < 0.03). The plasma concentration of adiponectin, CRP, glucose, HDL-cholesterol and total cholesterol/HDL-c ratio levels were not different. These results indicate that lifestyle associated with high intensity and high volume exercise induces changes favourable in the lipid profile and PAI-1 levels and may reduce risk cardiovascular diseases.

## Introduction

The several studies have consistently shown that low levels of plasmatic high-density lipoprotein (HDL) and high levels of low- and very low-density lipoprotein (LDL and VLDL, respectively) are linked with a sedentary lifestyle and are strong predictor to cardiovascular disease [1-5]. In addition, many of these diseases associated to sedentary lifestyle are also characterized by alterations in pro-inflammatory markers in the plasma [6,7]. These inflammatory process include increase in plasma the pro-inflammatory cytokines as tumor necrosis factor alpha (TNF-α), plasminogen activator inhibitor type-1 (PAI-1), C-reactive protein (CRP), interleukin 6 (IL-6) and reduction of anti-inflammatory cytokines as interleukin 10 (IL-10) and adiponectin [8-11].

The plasmatic PAI-1 and CRP levels are also strongly related to cardiovascular risk factors, increasing its levels leading of arising the hypertension, high triglyceride levels and likely obesity [6,12-16]. It is now established that reduction in the plasma to adiponectin and increased PAI-1 and CRP levels play a role in the maintenance of an inflammatory state and in the development of cardiovascular disease [4]. However, it is much less known if these pro- and anti-inflammatory markers are related to the lipoproteins and cholesterol levels in plasma. While competitive exercise training programs (characterized by moderate/high intensity and long duration) seem to reduce the LDL and total cholesterol and to increase the HDL concentration, evicting the progression or appearance of inflammatory atherosclerotic process [6], it remains slightly unknown if highly trained athletes have lower pro-inflammatory and higher anti-inflammatory markers than sedentary subjects.

Therefore, the objective of this study was to compare plasma levels adiponectin, PAI-1, CRP, and lipoprotein fractions between sedentary subjects and highly trained athletes. We hypothesised that individuals that performed high amount of exercise (moderate/high intensity and long duration) have improved lipid profile and inflammatory factors levels in relation sedentary subjects.

## Methods

### Subjects

Fourteen healthy men and non-smoking participated in this study. The subjects were sedentary lifestyle (n = 7) [age 28.6 (6.9) years, height 174.0 (0.04) cm, weight 75.6 (10.2) kg] and highly trained athletes in cycling (n = 7) [age 29.8 (5.7) years, height 177.0 (0.06) cm, weight 74.7 (4.4) kg]. The anthropometric parameters (height and body weight) were measured and standardized using Lohman's protocol [17]. The physical and training characteristics of both groups are described in Table 1. The benefits and risks were explained before written consent was obtained. The study procedures were previously approved by the Ethics Committee of the Federal University of São Paulo. Non-inclusion criteria were: identified genetic, metabolic or endocrine disease, previous drug utilization, time exercise training program or non-exercise.

**Table 1:** The height, body mass, and body mass index the both groups and the weekly exercise intensity, frequence, volume and time experience by highly trained athletes group.

Subjects	Sedentary	Highly Trained Athletes
Height (cm)	174 ± 0.05	177 ± 0.06
Body mass (kg)	75.6 ± 10.2	74.7 ± 4.46
BMI (kg/m^2^)	24.9 ± 2.54	23.9 ± 1.54

Experience exercise training (years)		8.86 ± 2.73
Frequence exercise (time for week)		4.43 ± 1.27
Volume exercise (hours for day)		2.14 ± 0.62
Intensity exercise (VO_2max_)		50-100%

Results are expressed as mean value ± SD.

VO_2max _test was performed of athletes in particular clinics. The data was sending for us analyzed. All data frequence, volume and intensity exercise were related by technical professional this athletes. The athletes remain 48 h without exercise.

### Blood sampling and analysis

A catheter was inserted in a brachial vein for drawing venous blood samples. The blood samples (10 ml) were immediately transferred into two 5-ml vacutainer tubes (Becton Dickinson, BD, Juiz de Fora, MG, Brazil) containing EDTA for plasma separation. The tubes were centrifuged at 3000 g for 15 minutes at 4°C, and plasma samples were stored at -80°C until analysis. Triglycerides, HDL cholesterol, and total cholesterol were assessed through commercial enzymatic kits (Labtest^®^, São Paulo, Brazil). LDL cholesterol was calculated according to Friedewald et al. [18] and LDL-oxidized was calculated according to Tsimihodimos et al [19]. Plasma glucose concentration was analysed using the enzymatic colorimetric method (Biotécnica, São Paulo, Brazil). PAI-1 and adiponectin were assessed through commercial kits (R&D systems^®^, São Paulo, Brazil). CRP was assessed through commercial kit (Bioclin^®^, São Paulo, Brazil).

### Statistical analyses

The data distribution was previously checked by the Bartlett's test for equal variances, and the data are reported as means and standard deviation. The differences in the plasma parameters among groups were accessed by unpaired t test. The Pearson correlation coefficient was calculated to assess the relationship between variables. The analysis was carried out using GraphPad Prism (version 5.00) software, and the significance level was set at p < 0.05.

## Results

The subjects have similar height, body mass, and body mass index (table 1). The weekly exercise intensity, frequence, volume and time experience by highly trained athletes groups are also showed in Table 1.

Table 2 show plasmatic lipid profile, glucose, PAI-1, adiponectin, and CRP levels for the two groups. Was observed less plasmatic levels the Total cholesterol (p < 0.001), LDL-c (p < 0.01) and TG (p < 0.038) in highly trained athletes than in sedentary group. Plasmatic PAI-1 level was significantly lower (p < 0.0001) in highly trained athletes when compared with sedentary group. The plasmatic adiponectin (p < 0.88), CRP (p < 0.93), glucose (p < 0.10), LDL-oxidized (p < 0.09), HDL-c (p < 0.32) concentrations and Total Cholesterol/HDL-c ratio (p < 0.49) were not significantly different between the groups.

**Table 2:** Lipid profile, glucose, PAI-1, adiponectin, and CRP levels in the sedentary and highly trained athletes groups.

Subjects	Sedentary	Highly Trained Athletes
TG (mmol/L)	1.40 ± 0.62	0.94 ± 0.45*
Cholesterol (mmol/L)	3.98 ± 0.27	2.84 ± 0.66***
LDL-c (mmol/L)	2.38 ± 0.16	1.50 ± 0.80**
LDL-ox (mmol/L)	1.37 ± 0.67	0.71 ± 0.42
HDL-c (mmol/L)	1.31 ± 0.23	1.15 ± 0.35
TC/HDL-c ratio	3.09 ± 0.51	2.72 ± 1.28
Glucose (mmol/L)	4.26 ± 0.23	4.45 ± 0.06
PAI-1 (ng/mL)	18.7 ± 2.00	4.50 ± 1.71***
Adiponectin (ug/L)	14.1 ± 3.48	13.73 ± 5.91
CRP (g/L)	2.83 ± 0.27	2.82 ± 0.25

Results are expressed as mean value ± SD. * p < 0.05; ** p < 0.01;

*** p < 0.001 significantly lower than Sedentary group.

**Figure 1:**
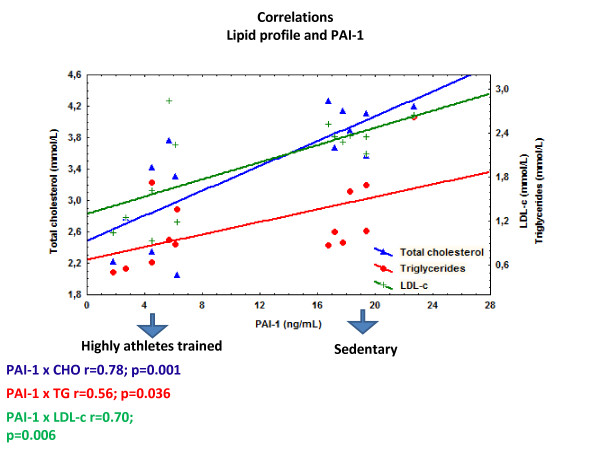
**Correlation between PAI-1 and total cholesterol level, PAI-1 and LDL-c level and PAI-1 and TG level (Figure 1)**.

Was found a correlation positive between plasmatic PAI-1 and total cholesterol level (r = 0.78, p = 0.0009). The plasma PAI-1 level was also positively associated with LDL-c (r = 0.69, p = 0.006) and TG (r = 0.56, p = 0.03). All correlation is showed in Figure 1. No significant correlations were found between PAI-1 and any other variable. No correlations were found between adiponectin and CRP with any variable (p > 0.05). The volume and exercise frequency of the highly trained athletes group was inversely associated with TG concentration (r = -0.76 and -0.77, respectively; p < 0.05; *data not showed*).

## Discussion

The results of the present study indicate that, highly trained athletes subjects that perform high amount of the exercise shown lower plasmatic lipid profile and PAI-1 levels than sedentary subjects. In addition, the plasma total cholesterol, LDL-c and TG concentration were positively correlated with pro-inflammatory PAI-1 levels.

The data of the present study confirm previous findings that subjects trained have lower plasmatic TG, LDL-c and total cholesterol concentration than sedentary subjects [20,21]. Tsekouras et al [22] examined the effect of high intensity intervals of aerobic training on VLDL-TG secretion in men. They observed that subjects who had trained by running on the treadmill for 8 weeks at 90% VO_2peak _had a reduced rate of VLDL-TG secretion, suggesting that subjects that perform high intensity exercise induced changes in plasmatic lipid profile. Moderate chronic exercise also induces an augment of lipase lipoprotein gene expression and activity, both in the skeletal muscle and the adipose tissue [23,24], resulting in decreased plasma TG content and liver VLDL output [25,26]. Additionally, aerobic exercise increase the activity of lecithin:cholesterol aciltransferase (L-CAT), the enzyme responsible for cholesterol ester transfer to HDL-c and reduces the activity of the plasmatic cholesterol ester transfer protein (CETP), the enzyme responsible for transferring the ester in HDL cholesterol to other lipoproteins [27-31]. These alterations may lower the concentrations of total cholesterol and LDL-c in the plasma through the exchange of cholesterol ester between tissues and lipoproteins to HDL-c [32].

The plasmatic PAI-1 levels were less in highly trained athletes in relation to sedentary subjects, as showed in Table 1. The fibriolytic system is a proteolytic enzyme system with many physiological functions of which degradation of fibrin deposits in blood vessels is the best known and possibly the most important. Studies have indicated that reduced fibrinolytic capacity, mainly due to elevated plasma levels of PAI-1, may have pathogenetic importance in myocardial infarction, particularly in patients with hypertriglyceridemia [33]. Páramo et al [4], observed a significant reduction in PAI-1 activity after aerobic regular long-term (9 months duration) indicating that exercise training had beneficial effect on fibrinolysis. Stratton et al [34] also showed that exercise training enhances fibrinolysis in healthy older men by increasing resting levels of tissue plasminogen activator and decreasing PAI-1 activity. The results of the present study indicated that high amount of exercise reduces PAI-1 levels in plasma.

Sedentary lifestyle increases the risk of developing cardiovascular disease and diabetes [1], and many other diseases which are linked to the inflammatory markers in the plasma [7]. Patients with elevated plasma cholesterol and with coronary atherosclerosis have increased plasma levels of PAI-1 [35]. Some studies indicate that this may be a consequence of the endothelial dysfunction caused by high plasma lipoprotein levels, and it is assumed that this is one of the principal causes by which these patients are at higher risk for myocardial infarctions [36]. The results of the present study comparing highly trained athletes with sedentary subjects indicates that high amount of exercise exert an important protector effect for reduced both lipid profile and PAI-1 levels.

A positive and fairly strong relationship between triglycerides, LDL, total cholesterol and PAI-1 levels in plasma is of particular interest, since it raises the possibility that hypertriglyceridemia is connected with a predisposition to thrombosis through a coexisting increase in PAI-1 concentration [33]. Jovin et al [35] related that, plasma LDL can induce an increased release of PAI-1 by endothelial cells into the vessel lumen and can contribute to the release of PAI-1 into the subendothelial space and thus to the process of atherosclerotic plaque remodelling and rupture. The significant correlations found in the present study between PAI-1 and TG, LDL and total cholesterol levels suggest that the elevated pro-inflammatory process in sedentary group may be due to the elevated values of the lipids in plasma.

In present study, no differences were verified in relation plasmatic CRP and adiponectin levels between sedentary and highly trained athletes. Changes in plasmatic CRP and adiponectin levels are more observed in weight loss interventions [5,6,37,38]. These data indicate that these adipokines may not be good indicators of processes linked to the increase in the lipid profile in subjects with sedentary lifestyle.

The limitations of this study include the relatively small numbers of subjects in each of the groups and the lack of subjects with cardiovascular and other diseases in which the changes in variables are assessed. Future studies should address the ability of exercise training to alter fibrinolytic variables in these groups and should also evaluate the effects of longitudinal training protocols.

In summary, our results indicate that sedentary subjects present a favourable state leading to hypertriglyceridemia and hypercholesterolemia accompanied by high PAI-1 levels. In contrast, highly trained athletes performance and high amount of the exercise reduced greatly the possibility of appearance of cardiovascular diseases.

## Competing interests

The authors declare that they have no competing interests.

## Authors' contributions

FSL, JCR, AELS, HAS, ECC, MCS, ARD, LMO and RVTS participed the sample collected, assess samples, design of the study and performed the statistical analysis, and writing of paper. All authors read and approved the final manuscript
